# Descriptive Study of Transgender Youth Receiving Health Care in the Gender Identity Program in Southern Brazil

**DOI:** 10.3389/fpsyt.2021.627661

**Published:** 2021-03-04

**Authors:** Bianca Machado Borba Soll, Anna Martha Fontanari, Angelo Brandelli Costa, Ítala Chinazzo, Dhiordan Cardoso Silva, Fernanda Guadagnin, Silzá Tramontina, Maria Inês Rodrigues Lobato

**Affiliations:** ^1^Programa de Identidade de Gênero, Departamento de Psiquiatria e Medicina Legal, Hospital de Clínicas de Porto Alegre (HCPA), Universidade Federal do Rio Grande do Sul (UFRGS), Porto Alegre, Brazil; ^2^Programa de Pós Graduação em Psicologia, Pontifícia Universidade Católica do Rio Grande do Sul (PUCRS), Porto Alegre, Brazil

**Keywords:** gender incongruence, gender dysphoria, transgender youth, healthcare service specialized in gender, gender-affirmative treatment model

## Abstract

Since 2014, the Gender Identity Program (PROTIG) of Hospital de Clínicas de Porto Alegre (HCPA) has been assisting transgender youth seeking gender-affirmative treatment offered at a public health-care service specializing in gender in southern Brazil. This article aims to analyze sociodemographic and clinical data regarding the diagnoses of gender dysphoria and gender incongruence, psychiatric comorbidities, and clinical aspects of a sample of transgender youths seeking health care in the gender identity program. The research protocol consisted of a survey of the data collected in the global psychological evaluation performed at the health-care service for youths diagnosed with gender incongruence and their caretakers. Participating in this research were 24 transgender youths between 8 and 16 years old with diagnostic overlap of gender dysphoria [*Diagnostic and Statistical Manual of Mental Disorders*, Fifth Edition (DSM-5)] and gender incongruence [*International Classification of Diseases, 11th Revision* (ICD-11)] and 34 of their caregivers. Of the young people, 45.8% were positive for some psychiatric comorbidity throughout their lives, with almost half (45.4%) having two or more psychiatric comorbidities in addition to gender dysphoria. The mental health professionals comprising affirmation care teams face the challenge of adapting the care protocols to the uniqueness of each demand by developing individualized forms to promote healthy development. This can be done by focusing not only on medical and physical interventions for gender affirmation but also on the promotion of mental health and general emotional well-being. Thus, the gender affirmation model, which advocates for global assessment and personalized guidance, proved to be adequate. Nevertheless, access to multidisciplinary health services specializing in gender is essential for promoting the general well-being of the population of transgender youth.

## Introduction

In recent years, there has been an increase in the seeking of assessment at specialized services for gender identity for youths who self-define as transgender people, requiring actions that promote the qualification of professionals focusing on this age group and technical protocols aiming at assistance standardization (1–3). The term *transgender* is a popular expression to include a broad spectrum of gender expressions (gender variance or gender non-conformity) that have been identified in our current society, while *cisgender* conceptualizes people who do not have a gender identity variant of the sex designated at birth. These expressions are not scientific terms and do not appear in any current diagnostic manual (4).

Among different possible care models for this population segment (transgender youth), what has been widely discussed and implemented today is the so-called “affirmative gender model,” which starts from the conceptual premise of the existence of different expressions and gender identities in society and the concept that each individual can be encouraged to discover his or her own expression, if he or she has a suitable environment for it, thus enhancing his or her emotional well-being (5). Because it is an approach that values the uniqueness of each experience and the context in which the young person is inserted, affirmative gender practice involves careful assessment and considers family dynamics, psychological profiles, the presence of diagnostic criteria for gender dysphoria (GD)/gender incongruence (GI), possible secondary gains, and the occurrence of clinical and/or psychiatric comorbidities (6). It should be noted that this care model also considers the complexity of factors that involve the specific stage of development (transition from childhood to adulthood), such as the age of legal majority, which can differ from country to country (7), the freedom or not of gender expression in the individual's social group and the assistance support that is available. Despite being a relatively new approach and still lacking scientific research, it is not limited to assessing whether young people should simply start the medical gender-affirmative treatments available for them but focuses on the global assessment of development (8).

Recent research with groups of transgender youth who access specialized health-care services has shown a high level of vulnerabilities in mental health when compared with cisgender groups of the same age, including suicide risk, use of psychoactive substances, anxiety disorders, depression, eating disorders, and autism spectrum disorders (9–18). Another factor that interferes with the well-being of young transgender people is the high level of violence to which they are exposed. Another factor is that violence against transgender people is often perpetuated by people known to the victim, including family members (19–21). It is known that the transgender population is systematically exposed to social stigma, prejudice, and rejection (minority stress), rendering it vulnerable to the emergence of psychiatric symptoms, and this factor is in isolation is possibly the main factor responsible for the psychological suffering caused in this population (22, 23). However, there is evidence that this fact can be strongly neutralized by simple access to specialized assistance (24, 25).

In Brazil, anyone who meets the criteria for GI [*International Classification of Diseases, 11th Revision* (ICD-11); WHO] has access to public health services for gender-affirmative treatment. According to a resolution of the Federal Council of Medicine (resolution CFM 2265/2020)—the document that regulates the attendance of gender-affirmative care in the Brazilian Unified Health System (SUS)—young people who self-define as transgender must be attended by a specialized multidisciplinary team and can have access to hormonal treatment when they reach 16 years of age if they desired and if the youth has the consent of the legal caregiver. Treatment with hormonal blockers intended for prepubescent children can be administered when there is a demand, only for research purposes. Even though there is a regulation of health services, there are many social barriers to this population for accessing these health-care services. Specialized health services remain extremely limited, and the wait for access to the health system in Brazil is long: 9 years on average (26). Therefore, it is very important for people to have access to health services and vulnerability prevention. The quality of interventions is associated with how early such service is established, preferably before adolescence (27, 28).

Thus, due to the lack of studies in our country regarding this population profile, this article aims to describe, from sociodemographic, clinical, diagnostic, and comorbid points of view, a sample of youths self-defining as transgender people who sought specialized assistance for gender affirmation in a public hospital in southern Brazil.

## Methodology

This research was approved by the Ethics Committee of the Hospital de Clínicas de Porto Alegre (HCPA) (protocol no. 140275). All of the participants, both young people and their caregivers, were informed of the purposes of this research and then invited to participate. All of the subjects provided written informed consent.

### Participants

A total of 24 young people participated, aged 8 to 16 years, who met the diagnostic criteria for GD [*Diagnostic and Statistical Manual of Mental Disorders*, Fifth Edition (DSM-5)]/GI (ICD-11). All of the families of the young people were invited to participate to complement the history data and to participate in therapeutic planning. Twenty-one mothers (aged between 27 and 61 years) and 13 fathers (aged between 30 and 64 years) accepted the invitation. The exclusion criterion was a lack of a GI diagnosis (ICD-11).

### Procedures

The research protocol consisted of the systematization of the data collected in the global psychological evaluation performed during the initial health-care encounter of the young people and family members who sought help at the Gender Identity Program (PROTIG) at the HCPA. For the present study, the following techniques were considered: psychological clinical interview, psychiatric evaluations, structured interviews, cognitive testing, and psychopathology evaluation instruments—applied to each individual and to the caregiver present throughout the entire care.

#### Psychological Clinical Interview

In this evaluation, made with young people and with caregivers, three interviews were conducted by a childhood and adolescence psychologist aiming at (1) the establishment of a therapeutic alliance; (2) evaluation of emotional development; (3) evaluation of family dynamics; (4) evaluation of the social support network; (5) evaluation of knowledge about legal rights; and (6) evaluation of the level of self-recognition and expression of the youth's gender identity.

#### Psychiatric Evaluation

This evaluation, conducted by a psychiatrist specializing in childhood and adolescence, is conducted on young people and caregivers. The number of meetings varied according to individual needs and depended on the presence of psychiatric morbidities, and they were involved in monitoring and evaluating the therapeutic response to drugs.

#### Structured Interview

For this interview, a questionnaire used in the Brazilian field study for the ICD-11 (WHO) was applied, which overlapped the diagnostic criteria for GI (ICD-11-2018) with the diagnostic criteria of DG (DSM-5-2013) (26, 29, 30). The survey instrument, based on participants' reports, evaluated a particular period of time, that is, when they first became consciously aware that they might be trans (incongruence between one's experienced gender and assigned sex). For this research, three clusters from the questionnaire were used: (1) diagnostic criteria; (2) degree of psychological distress; and (3) loss of social functioning (here represented by school dropouts due to gender incongruity). The intensity of emotion, as well as the intensity of discomfort with sexual characteristics, was assessed using a Likert scale ranging from 1 (very little) to 5 (very much). This interview was administered by a trained psychologist and was aimed at young people, but the questions can also be adapted to be answered by caregivers. If it made the participants feel more comfortable, the caregivers could participate.

### Measure

#### Wechsler Intelligence Scale for Children

In this evaluation, made with young people and performed by a psychologist trained in its application, the validated version for the Brazilian population of the Wechsler Intelligence Scale for Children (WISC-VI) scale was used, consisting of 15 subtests (10 main and five supplementary) and four indices, namely, the Verbal Comprehension Index, the Perceptual Reasoning Index, the Working Memory Index, and the Processing Speed Index. In total, these scales comprise the Full-Scale IQ (FSIQ) (31).

#### Schedule for Affective Disorders and Schizophrenia for School-Age Children Present and Lifetime Version

This semistructured interview is conducted with young people and with caregivers and performed by a psychologist and/or psychiatrist trained in its application. The validated version for the Brazilian population of the Kiddie-Sads scale was used to estimate the presence of psychopathology and diagnostic status over the lifetime of the youths according to the DSM-IV criteria (32).

### Data Analysis

Descriptive and statistical analyses were performed using SPSS software (version 21.0). For the present study, 41 variables were selected from the database. Categorical variables (sex designated at birth, gender identity, school evasion, commencement of social gender transitions, use of hormones, etc.) were expressed by frequencies and percentages. The ordinal variables (age, years of study, age of parents, age of perceived gender incongruity by participant, age of perceived gender incongruity by participants' parents, age of social transition, FSIQ, etc.) were expressed through means and standard deviation.

## Results

### Sociodemographic Data

The sample consisted of 24 young people (average age = 13.88 ± 2.455 years) and 34 of their caregivers, of whom 21 were mothers (average age = 43 ± 9 years) and 13 were fathers (average age = 45.8 ± 9.5 years). Most of the caregivers (74.3%) were employed at the time of the evaluation, and 35.3% had college degrees. Of the youths, half had the sex designated at birth as female and identified with the male gender (50%), and the other half had the sex at birth designated as male and identified with the female gender (50%).

Table 1 shows the sociodemographic data of these young people.

**Table 1:** Sociodemographic data referring to the studied youth population.

	Age Mean (SD)^**^	**Public school*N* (%)**	**Private school *N* (%)**	School evasion^*^N (%)	Years of study Mean (SD)^**^
Total(24)	13.88 (2.45)	15 (62.5)	9 (37.5)	8 (33.3)	8 (2.432)
Female gender identity(12)	13.83 (2.73)	6 (50)	6 (50)	3 (25)	8.5 (2.431)
Male gender identity(12)	13.92 (2.28)	9 (75)	3 (25)	5 (41.7)	7.5 (2.431)

*SD, standard deviation*.

* *Due to gender incongruence*.

** *Mean in years*.

### Diagnostic Confirmation

All of the participants had the diagnosis of GI (ICD-11).

### Gender Perception and Social Gender Transition

Most of the participants (79.2%) had already started social gender transitions when they sought gender-affirmative health care.

Table 2 shows the mean age that the subject realized that his or her gender was incongruous with the sex designated at birth (age of perceived gender incongruity by participant); the mean age at which the parents perceived that their daughter/son exhibited some socially unexpected gender behavior for the assigned sex (age of perceived gender incongruity by participants' parents); and the mean age at which these youths started the social transition of gender roles (age of social transition).

**Table 2:** Gender perception and social gender transition.

	**Male gender identityMean (min/max)**	**Female gender identityMean (min/max)**	**Total (24)** ** Mean (min/max)**
Age of perceived gender incongruity by participant	9.5 (5–15)	9 (5–13)	9.25 (5–15)
Age of perceived gender incongruity by participants' parents	5.9 (2–12)	6.5 (2–14)	5.83 (2–14)
Age of social transition	13.29 (10–15)	12.83 (7–16)	14 (7–16)

### Discomfort With Sexual Characteristics

Only two young people (8.3%) used hormones without medical supervision before entering gender-affirmative health care. Regarding discomfort with sexual characteristics, 58.3% (*n* = 14) reported feeling strong or very strong discomfort with the genitals, and only one (4.2%) reported not feeling any discomfort with the genitals. Only two subjects (8.3%) reported not feeling any discomfort with the breasts; however, 75% reported that their discomfort was strong or very strong. Regarding the tone of the voice, half (50%) reported not feeling discomfort, and seven subjects (29.2%) reported strong or very strong discomfort with the tone of voice.

Table 3 shows the level of discomfort with sexual characteristics (discomfort about his or her sexual anatomy or anticipated secondary sex characteristics) divided by gender identity.

**Table 3:** Level of discomfort with sexual characteristics.

**Discomfort with sexual characteristics**	**Male gender identity** ***N*** **(%)** **Total 12**		**Female gender identity** ***N*** **(%)** **Total 12**	
Genitals	No discomfort 0 (0)	Strong/very strong 3 (25)	No discomfort 1 (8.3)	Strong/very strong 11 (91.7)
Chest	No discomfort 1 (8.3)	Strong/very strong 11 (91.7)	No discomfort 1 (8.3)	Strong/very strong 7 (58.3)
Voice	No discomfort 7 (58.3)	Strong/Very strong 4 (33.3)	No discomfort 5 (41.7)	Strong/very strong 3 (25)

### Psychological Distress and Feelings of Rejection due to Gender Incongruence

Slightly more than half of the participants (54.2%) claimed to have felt rejected because of their gender incongruity. All of the subjects (100%) said they felt significant distress (sadness and anxiety) associated with their gender incongruity in an intense fashion and for a period longer than 6 months. Of these cases, 91.7% (*n* = 22) associated this psychological distress with the conflict between having a biological body incongruent with the gender of identity, and two of them, 8.3%, associated their psychological distress with reactions of social stigma and prejudice. Of those who had school evasion (*n* = 8) (see Table 1), 37.5% claimed that it was due to the conflict between having a biological body incongruent with the gender identity, and 62.5% claimed that it was due to social reactions regarding their expressed gender.

Table 4 shows the occurrence of psychiatric morbidities present in the sample and the mean of the FSIQ.

**Table 4:** Occurrence of psychiatric vulnerabilities and diagnosis status in the present and/or past lifetime and full-scale IQ.

**Psychiatric vulnerabilities**	**Male gender identity (*N*)**	**Female gender identity(*N*)**	**Total** ***N* (%)**
FSIQ (N)(Mean; SD)	11(88.55; 14.2)	12(93.3;13.2)	23 (91; 13.6)
K-SADS-PL Positive	6	5	11 (45.8)
Alcohol abuse	–	1	1 (4.16)
Abuse of psychoactive substances	–	2	2 (8.33)
Major depression	1	3	4 (16.6)
Dysthymia	1	3	4 (16.6)
Oppositional defiant disorder	4	2	6 (25)
Conduct disorder	1	–	1 (4.16)
Anorexia	1	–	1 (4.16)
Bulimia	1	–	1 (4.16)
Autism spectrum disorders	–	–	–
Suicide attempt	–	3	3 (12.5)
Psychiatric hospitalizations	–	3	3 (12.5)^*^
Gender dysphoria	12	12	24 (100)

*K-SADS-PL, Schedule for Affective Disorders and Schizophrenia for School-Age Children Present and Lifetime Version; FSIQ, Full-Scale IQ*.

* *Due to suicide attempt*.

Of the young people, 45.8% were positive for some psychiatric comorbidity throughout their lives, and of these people, almost half (45.4%) had some diagnostic co-occurrence; that is, they were positive for two or more psychiatric diagnoses in addition to DG/GI. Of the youths who tested positive for Schedule for Affective Disorders and Schizophrenia for School-Age Children Present and Lifetime Version (K-SADS-PL), 81% (10) had a qualitative FSIQ less than the average (<89 composite points).

## Discussion

The DSM-5 classifies the presence of psychological distress or social dysfunction due to gender incongruity as a required criterion for the diagnosis of GD. The ICD-11, conversely, does not require the presence of the criterion “psychological distress” for the diagnosis of GI since it understands that transgender people might not present this type of suffering experience. In our study, most of the youths associated this psychological distress with the conflict regarding gender incongruity itself; thus, it came as a consequence of the emotional conflict of realizing the incongruity of their biological bodies with their gender identity, and a minority associated psychological distress with the social stigma and prejudice resulting from their expressed gender variance. However, stigma and prejudice were perceived as the main reasons for school evasion. Many schools are still aligned with rigid structures of binary gender roles, such as wearing uniforms and toilets divided by sex, making transgender youth inclusion difficult. This result provides evidence that the stigma and prejudice suffered by these people directly affect their social functionality. Other studies have emphasized the negative social and emotional impacts of stigma and prejudice on the lives of transgender people (19, 20) and have reinforced the need for interventions in different social structures. Thus, positive public policies can guarantee that transgender people have their rights preserved and that their gender expression is not a limiting factor or seen as harmful in their social spheres.

Discomfort with existing or anticipated sexual characteristics is one of the main factors why young people seek gender-affirming health-care services. Discomfort with the genitals, the chest, and the voice are still the most reported; however, the results showed a personal and gender identity variation regarding the level of this discomfort (see Table 3). Regarding medical treatment of gender affirmation, a minority (8.3%) started using hormones without adequate health monitoring. In a similar study conducted with a group of adult individuals diagnosed with GD in health care at a Brazilian specialized gender service, at least one affirmative gender intervention without medical monitoring (use of hormones and/or aesthetic procedure) was found in 62.1% of individuals (26). These contrasting findings reinforce the need to offer specialized gender care services to transgender youth to avoid possible iatrogenic procedures. Regarding aspects of gender transition that do not involve medical interventions, most young people arrived at the affirmative care service with the social transition already started. Social transition refers to the process by which someone advances his or her experience in the gender role with which he or she identifies, affirming his or her gender identity, such as using a name or pronoun and/or wearing clothes that align more with gender identity than with sex assigned at birth. Currently, in most cases, this initiative seems to be attributed to a decision by the family nucleus that does not use specialized support to initiate a social gender transition that does not involve medical procedures. The results show that the beginning of the social transition is much later than the age of the subject's perception of their gender and of the age at which parents perceive the gender incongruity of their son or daughter (Table 2). Gender perception variables were raised using a structured interview that directly asks the subject's age of perception. This is combined with the psychological evaluation data in which the young people and their caregivers reported perceiving the expression of a deeply essential gender identity discordant from assigned sex. Researchers have maintained that there is the possibility of recognizing a stable transgender identity from childhood. They show that, in general, the onset age of the first behavioral manifestations occurs during preschool years, younger than five (33–37). It has been increasingly accepted among many gender theorists and practitioners that an individual's gender is evolutionary across the life span. Gender-affirmative care places substantial significance on a child's understanding of their own gender and allows the youth, and their knowledge of their gender, to lead the way to interventions. This includes the phase in which the young people and their families feel safe commencing the social gender transition (34).

In the present study, a high index of impairment in cognitive function was found in people who had a mental health diagnosis in addition to GI/GD. It is known that it is common to find the co-occurrence of intellectual disabilities and psychiatric disorders in children and adolescents (38, 39). Together with gender issues, mental disorders and intellectual disabilities are substantial public health problems. This fact has proved to be another challenge for health teams that provide health prevention strategies, adherence to treatment, and improvement in quality of life in all spheres, both personal and social, requiring individualized interventions. During the transition from childhood to adulthood, addressing the changes in a body that cannot be identified is a highly emotionally demanding task in itself and can certainly be detrimental to psychological, intellectual, and social development in transgender people.

The removal of transgender identities from the mental health category in the ICD-11/WHO is an important milestone in the attempt to not stigmatize gender identities (29). However, our study revealed that almost half of the sample was diagnosed with some psychiatric comorbidity throughout their lives. The above findings are in line with the results of other centers serving transgender youths in different countries and cultures (40, 41). Additionally, these results are close to those found in the population of transgender adult people who sought specialized gender health-care services in Brazil, in which 42.7% of the sample had some psychiatric comorbidity (42). These findings emphasize the need for the presence of a qualified mental health team in gender-affirming clinical care. In contrast, positive findings regarding mental health in recent studies have shown a reduction in psychopathology after simple access to health-care services (24, 25, 43, 44).

However, clinical practice with transgender youth challenges health teams to develop care protocols that focus on singular interventions that cover the different social structures that make up the individual's environment, as well as specific and appropriate interventions for each situation regarding the demand for medical treatment of gender affirmation and social transition. In light of the complexity of serving this population, it is necessary to invest in multidisciplinary teams that develop individualized ways to promote healthy development. Thus, the gender affirmation model, which advocates for a global assessment and personalized guidelines, proved to be adequate for the clinical follow-up of young people who sought specialized gender health-care services, therefore, focusing on the promotion of mental health, general emotional well-being, and recognition of legal and social rights in different spheres of the environment and not only medical procedures and physical interventions of gender affirmation.

## Conclusion

The entire conduct of the gender-affirmative clinical process must be undertaken from a scientific/practical perspective (from evaluation to intervention) so that it can support best ethical practices and facilitate the selection and monitoring of therapeutic efficacy. Nevertheless, access to multidisciplinary health services specializing in gender is essential for promoting the general well-being of the population of transgender youth.

### Limitations

The main limitation present in the study is the sample size. The specialized gender services for minors are still recent in Brazil. In addition, there are many social barriers to this population for accessing health-care services. This study analyzes the average of a group and cannot be used to characterize an individual within the group.

## Data Availability Statement

The original contributions generated for this study are included in the article/supplementary material, further inquiries can be directed to the corresponding author/s.

## Ethics Statement

The studies involving human participants were reviewed and approved by This research was approved by the Ethics Committee of the Hospital de Clínicas de Porto Alegre/HCPA (protocol no. 140275). Written informed consent to participate in this study was provided by the participants' legal guardian/next of kin.

## Author Contributions

BS, AB, and ML conceived of the presented idea. BS performed the research protocol. ST helped supervise the project. ÍC, DS, AF, and FG contributed to the interpretation of the results. All authors discussed the results and contributed to the final manuscript.

## Conflict of Interest

The authors declare that the research was conducted in the absence of any commercial or financial relationships that could be construed as a potential conflict of interest.
