# Corrigendum: Descriptive Study of Transgender Youth Receiving Health Care in the Gender Identity Program in Southern Brazil

**DOI:** 10.3389/fpsyt.2021.722544

**Published:** 2021-07-19

**Authors:** Bianca Machado Borba Soll, Anna Martha Fontanari, Angelo Brandelli Costa, Ítala Chinazzo, Dhiordan Cardoso Silva, Fernanda Guadagnin, Silzá Tramontina, Maria Inês Rodrigues Lobato

**Affiliations:** ^1^Programa de Identidade de Gênero, Departamento de Psiquiatria e Medicina Legal, Hospital de Clínicas de Porto Alegre (HCPA), Universidade Federal do Rio Grande do Sul (UFRGS), Porto Alegre, Brazil; ^2^Programa de Pós Graduação em Psicologia, Pontifícia Universidade Católica do Rio Grande do Sul (PUCRS), Porto Alegre, Brazil

**Keywords:** gender incongruence, gender dysphoria, transgender youth, healthcare service specialized in gender, gender-affirmative treatment model

In the original article, we neglected to include the funder *Fundo de Incentivo à Pesquisa e Eventos (FIPE) do Hospital de Cl*í*nicas de Porto Alegre*. The correct funding statement appears below.

**Funding**:

This study was financed in part by the Coordenação de Aperfeiçoamento de Pessoal de Nível Superior—Brasil (CAPES)—Finance code 001and Fundo de Incentivo à Pesquisa e Eventos (FIPE) do Hospital de Clínicas de Porto Alegre.

The authors apologize for this error and state that this does not change the scientific conclusions of the article in any way. The original article has been updated.

